# The Aging Kidney: Increased Susceptibility to Nephrotoxicity

**DOI:** 10.3390/ijms150915358

**Published:** 2014-09-01

**Authors:** Xinhui Wang, Joseph V. Bonventre, Alan R. Parrish

**Affiliations:** 1 Department of Medical Pharmacology and Physiology, School of Medicine, University of Missouri, Columbia, MO 65212, USA; E-Mail: xwxvb@mail.missouri.edu; 2 Renal Division and Biomedical Engineering Division, Department of Medicine, Brigham and Women’s Hospital, Harvard Medical School, Boston, MA 02115, USA; E-Mail: jbonventre@hms.harvard.edu; 3 Department of Medical Pharmacology and Physiology, School of Medicine, University of Missouri, Columbia, MO 65212, USA

**Keywords:** aging, acute kidney injury (AKI), chronic kidney disease (CKD), nephrotoxicity

## Abstract

Three decades have passed since a series of studies indicated that the aging kidney was characterized by increased susceptibility to nephrotoxic injury. Data from these experimental models is strengthened by clinical data demonstrating that the aging population has an increased incidence and severity of acute kidney injury (AKI). Since then a number of studies have focused on age-dependent alterations in pathways that predispose the kidney to acute insult. This review will focus on the mechanisms that are altered by aging in the kidney that may increase susceptibility to injury, including hemodynamics, oxidative stress, apoptosis, autophagy, inflammation and decreased repair.

## 1. Introduction

During the last century, human lifespan has increased substantially resulting in a substantial increase of elderly people over the next two decades [1]. Individuals aged 65 years or more represented 12.8% of US population in 2008 [2]. By 2030, the number of elderly people is expected to be 71 million, accounting for 21% of US population [3]. In fact, the elderly population is the most rapidly growing population segment in the western world [4]. It is estimated that by 2025, there will be over 800 million individuals over the age of 65 worldwide [5]. Thus, the study of age-dependent pathophysiology, and translation of these findings to the clinic, is a significant challenge for biomedical sciences in the 21st century.

Acute kidney injury (AKI), previously called acute renal failure (ARF) [6], is defined as an abrupt onset of renal dysfunction ranging from minor loss of function to failure [7,8,9]. AKI is a common clinical complication that develops in approximately 4%–7% of hospitalized patients each year and the prognosis can be poor [10,11]. While the mortality rates for AKI are decreasing, the mortality range remains from 20%–35% [12,13] and “nearly 2 out of 3 patients suffering from ARF will not be alive 90 days after the onset of ARF” [14]. Thus, AKI remains a significant public health problem. A relationship between AKI and the elderly has long been recognized [15,16,17]. In 1972, studies suggested that 23.8% of patients with AKI were over the age of 60 [15] and the following year it was shown that 13.7% were older than 70 [16]. In 1987, these percentages had risen to 76% and 46%, respectively [17]. The increasing prevalence of elderly AKI patients is supported by additional studies; Turney *et al.* [18] showed that the median age of AKI patients was 41.25 years in the 1950s and increased to 60.5 in the 1980s. In a study by Rosenfeld *et al.*, the average age of those who succumbed to AKI was 71.9 ± 8.8 years old, demonstrating the importance of the relationship between aging and AKI [17]. While this relationship has been established for four decades, interest in this area has dramatically increased and AKI in the elderly has been the subject of several recent reviews [19,20,21,22,23]. In this review, we will address the evidence that demonstrates the strong linkage between aging and AKI and the insight into mechanisms underlying this effect.

## 2. Aging and AKI: Clinical Evidence

In the past twenty-five years, a number of studies have associated aging with a higher risk for AKI [24,25]. Pascual *et al.* performed a study in Spain demonstrating that the incidence of AKI is 3.5 times higher in patients over 70 than those under 70; patients older than 80 years old were 5.0 times more likely to develop AKI [26]. Age above 65 years has also been shown to be an independent risk factor for AKI in a multinational, multicenter study [27]. Balardi and colleagues have shown that elderly patients (≥65 years) had ten times the incidence rate of AKI compared with those less than 65 years of age in Italy [28]. Xue *et al.* established age as a risk factor for AKI; the incidence of AKI was 1.9% in patients younger than 65 and rose to 2.9% in those older than 85 [13]. Data from a community-based cohort in California showed that the incidence of AKI not requiring dialysis was 79 per 100,000 person-years in patients younger than 50 and 3545 in patients over 80 [29]. Most recently, an increase in AKI in the elderly was seen following crush injury due to the earthquake in Wenchuan, China in 2008; compared to the resident population, the elderly patient with crush-related AKI was 2.6-fold higher than the younger patients [30]. Moreover, AKI that develops in the elderly is more severe and the patient is less likely to recover. Venkatachalam *et al.* showed that the percentage of elderly patients who did not recover renal function was 31.3% compared with 26% of younger cohorts [31]. Hospitalized AKI patients requiring dialysis are older than their counterparts who do not require dialysis (63.4 *vs.* 47.6 years) [32,33]. No increase in mortality has been consistently reported [17,24,34,35,36,37]; however, studies have suggested that mortality following AKI is increased in the elderly [15,38].

## 3. Aging and AKI: Experimental Models

A number of animal studies in the 1980s indicated that the aging kidney has a greater susceptibility to both ischemic and toxic injuries. Many of these studies used rats and it must be noted that due to the progressive nephropathy in senescent rats, they may not represent the optimal model to investigate xenobiotic-induced injury [39]. Beierschmidt *et al.* demonstrated an age-related increase in acetaminophen nephrotoxicity in male Fischer 344 rats, comparing rats at 2–4, 12–14 and 22–25 months of age [40]. Interestingly, baseline BUN, urine osmolality and urine volume were similar in all groups, suggesting that a major component of aging was increased sensitivity to insult as opposed to an overt loss of renal function. The nephrotoxicity of gentamicin is increased in the aging rat with no alteration in the pharmacokinetics of the antibiotic [41]. These findings were verified with female rats, but suggested that the lack of a relationship between the loss of renal function (decreased glomerular filtration rate (GFR)) and tubular injury (necrosis or casts) indicated that age-related changes reflected alterations in renal hemodynamics, rather than differences in the tubular susceptibility to injury [42]. In contrast, however, Miura *et al.* [43] demonstrated that slices of kidney from old rats were more susceptible to *in vitro* anoxia (100% nitrogen) than slices from young counterparts as assessed by organic anion transport in the proximal tubules, leading the authors to conclude that a component of the increased sensitivity to injury involves age-dependent alterations in the proximal tubules. Previous studies in our laboratory also showed similar results that renal slices from aged Fischer 344 rats fed *ad libitum*, but not aged caloric-restricted male animals, were more susceptible to ischemic injury (100% nitrogen) when compared with slices from young animals as assessed by histological and biochemical evaluations [44]. These *ex vivo* studies again demonstrated that the proximal tubular epithelial cells had an inherent increase in susceptibility to injury. Aged Wag/Rij rats (23–26 months) are more sensitive to tobramycin, an aminoglycoside antibiotic, as evidenced by tubular necrosis and urinary NAG levels [45]. More recent studies have extended age-dependent AKI models to the mouse. The aging (46–49 weeks) male C57Bl/6 mice exhibited prolonged elevation of plasma creatinine and greater mortality after bilateral renal ischemia-reperfusion (I/R) induced AKI compared to the young (8–10 weeks) [46]. Star and colleagues [47] developed a sepsis-induced AKI model by cecal ligation puncture (CLP) using aged (10.5–11 months) mice. Lipopolysaccharide (LPS) induced an increase in BUN and creatinine in the aged, but not young mice, setting the stage for the development of the more complex, clinically relevant CLP model. These results indicate that laboratory models recapitulate the clinical scenario of age-related AKI in humans and allow for the elucidation of specific mechanisms.

## 4. Aging and AKI: Causes

### 4.1. Chronic Kidney Disease (CKD)

**Figure 1:**
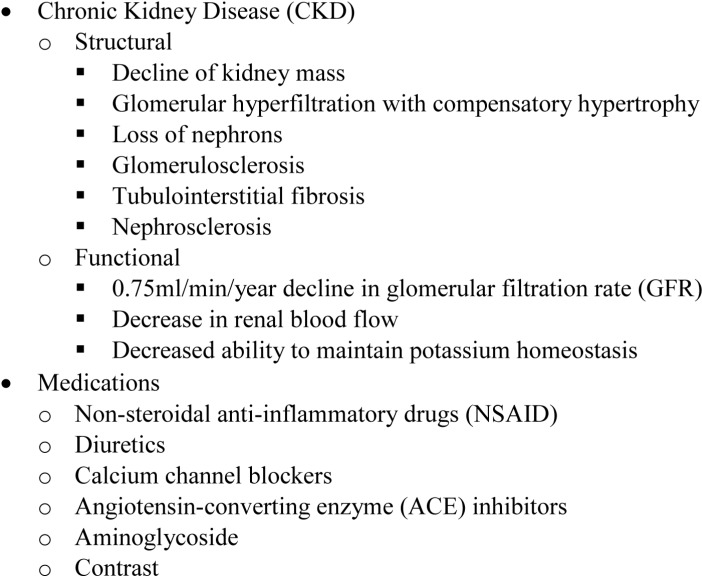
Causes of increased incidence of acute kidney injury (AKI) with aging.

It is well established that aging is associated with structural and functional renal changes (Figure 1) [48]. It has been stated that “with the possible exception of the lung, the changes in kidney function with normal aging are the most dramatic of any human organ or organ system” [49,50]. The normal kidney loses about 20%–25% of its mass during aging, with the loss involving both cortical glomeruli and tubules [51]. Age-dependent decline in kidney volume was not detectable by imaging, possibly due to the compensatory hypertrophy of functional nephrons [52,53]. In addition to nephron loss, glomerulosclerosis and tubulointerstitial fibrosis define the aging kidney [54,55]. There is also an increasing incidence of nephrosclerosis with aging from 2.7% for people aged 18–29 years to 73% for people aged 70–77 years [56]. Functionally, the aging kidney has a parallel decline in both glomerular and tubular function [57]. The seminal Baltimore longitudinal study demonstrated an average of 0.75 mL/min yearly decline in GFR in 254 men without hypertension or kidney disease [58]. A similar rate of decline (0.63 mL/min/year) was reported in a recent study based on 1203 living kidney donors [56]. The GFR loss rate is tripled in subjects over 40 as compared with those under 40 in the Baltimore study [58]. A more recent study in healthy Chinese people described similar results [59].

Approximately 35% of the elderly US population has stage 3 CKD [3] and an increasing number of elderly with CKD developed the end-stage kidney disease (ESKD) which requires dialysis [60]. There is a growing recognition that AKI most often occurs on a background of CKD [12,61]. Elderly patients who developed AKI on a background of CKD are less likely to recover from AKI and more likely to progress to more advanced stage or even ESKD which contributes to the higher mortality rate [62,63]. The hazard ratio of developing ESKD for patients with both AKI and CKD is 13.0 relative to those only with AKI [64]. The two-year mortality rate is higher for those with AKI and CKD (64.3%) than those with AKI alone (54.3%) [64]. On the other hand, AKI also predisposes patients to CKD after tubular regeneration due to inflammatory responses, paracrine stimulation of myofibroblasts, epithelial cell senescence, and loss of cellular plasticity, all of which promote a pro-fibrogenic phenotype [65,66].

### 4.2. Medications

Older individuals more commonly develop diabetes mellitus, hypertension, atherosclerosis, and heart failure, each of which can directly increase the risk of AKI [32]. These comorbidities can also increase the risk of AKI indirectly by leading to increased medication use (Figure 1) in elderly patients as compared with younger patients [63]. Around 20% of the episodes of AKI are induced by nephrotoxic drugs and the incidence of drug-induced nephrotoxicity leading to AKI among elderly in the hospital can be as high as 66% [67,68].

Potassium homeostasis is regulated, in part, by excretion of potassium into the urine via active secretion by principal cells in the collecting tubule; aldosterone is an important part of this control system. The direct aldosterone response to a potassium load is diminished in aging patients which increases vulnerability to hyperkalemia [69]. As a result, drug-induced hyperkalemia is more prevalent in aging patients [70].

AKI secondary to non-steroidal anti-inflammatory drugs (NSAIDs) is more common in the elderly [71]. More than 80% of patients with NSAID induced AKI are over the age of 60 [72]. In a study with patients aged 50–84 years, the relative risk (RR) for AKI was 3.2 in NSAID users and was increased dramatically when NSAIDs were used in combination with diuretics (RR 11.6) and calcium channel blockers (RR 7.8) [73]. The combination of NSAIDs and angiotensin-converting enzyme (ACE) inhibitors was also demonstrated to be associated with nephrotoxicity in elderly patients (>75 years) [74]; The incidence of aminoglycoside-induced nephrotoxicity is also elevated in the elderly [75,76] and increased injury in response to aminoglycosides in combination an ACE inhibitor in elderly patients has been reported [77]. While contrast-induced nephropathy (CIN) is a significant cause of AKI in hospitalized elderly patients [68,78], age *per se* may not be an independent predictor of contrast nephropathy [79]. It is expected that CIN will remain an important cause of AKI in the elderly due to the increased use of contrast media in this population [80].

## 5. Aging and AKI: Mechanisms

**Figure 2:**
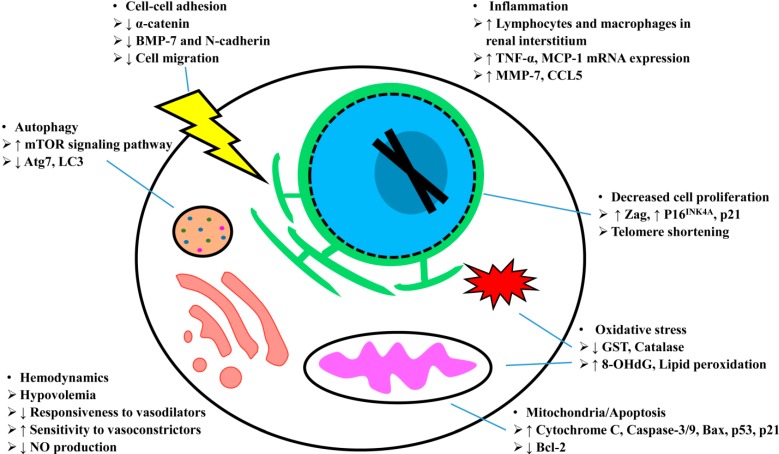
Mechanisms of increased incidence of AKI with aging.

The impact of kidney aging on pharmacokinetics has long-been recognized and is the subject of many reviews [76,81]. Importantly, the T_1/2_ of a number of drugs with potential adverse effects on the kidney, including NSAIDs and antibiotics, is increased in elderly patients [82]. As such, the increased nephrotoxicity of cephaloridine, a broad spectrum antibiotic, in aging male Fischer 344 rats (27–29 and 10–12 months compared to 2.5 months) is associated with increased serum and cortical concentrations of the antibiotic [83]. However, the cortical concentrations were similar in the 10–12 and 27–29 month rats, while proximal tubule dysfunction, as assessed by tubular transport, was worse in the older rats, suggesting that there is an increased sensitivity of the aging proximal tubular epithelium to injury. This highlights the fact that AKI in the elderly is multifactorial, involving a number of potential mechanisms that we will attempt to delineate (Figure 2).

### 5.1. Hemodynamics

Hypovolemia, both true (dehydration, bleeding, vomiting, diarrhea) and functional (cardiac impairment, liver disease, nephrotic syndrome) is common in elderly patients [84]. Until about the fourth decade, renal blood flow is maintained at approximately 600 mL/min. It then drops by approximately 10% every decade due to the activation of sympathetic nervous system, renin-angiotension-aldosterone system and vasopressin secretion [62,85]. The aging rat kidney is characterized by focal loss of peritubular capillaries spatially associated with tubulointerstitial injury [86,87], which contributes to chronic hypoxia in the aging kidney [88].

A role of nitric oxide (NO) in the increased sensitivity of the aging kidney to injury has been established. Reduced NO in peritubular capillaries in the aging kidney has been demonstrated which contributes to increased vasoconstriction, sodium retention, mesangial fibrosis and matrix production [86,89]. Increased I/R injury in aged (18 months) rats as compared to young (3 months) rats was associated with a dramatic increase in vasoconstriction in the aged rats. l-arginine, the precursor of NO synthesis, attenuated injury in aged rats to a greater extent than the young ones [90]. Taken together, these results suggest that the aging is associated with decreased NO levels which exacerbate I/R injury. Later studies demonstrated that NO levels, as well as acetylcholine-induced vasodilation, are reduced in the aging rat kidney [91]. These results were strengthened when it was shown that atorvastatin which enhances NO bioavailability in the aging rats afforded protection against I/R injury [92]. Another factor in the diminished NO-mediated responsiveness is the increased levels of the endogenous nitric oxide synthase (NOS) inhibitor, asymmetric dimethylarginine, in aging rats [93] and humans [94]. The increased nephrotoxicity of gentamicin has also been postulated to involve decreased NO production, as glomerular nitrate levels are decreased in aged rats (12 months) as compared to young controls [95]. In elderly patients (81–96 years), NO did not increase after vasodilatory stimuli [96].

In addition, the aging kidney has diminished responsiveness to vasodilators and increased sensitivity to vasoconstrictors [97]. Vasodilators, including pyrogen and acetylcholine [98], ANP [99], dopamine [100] and amino acids [100,101], have decreased functionality in the aging kidney. This decreased vasodilatation is coupled with increased vasoconstrictive response to sympathetic activation [102] or AngII [103]. Clinically, prolonged vasoconstriction due to contrast administration is associated with acute renal dysfunction in elderly patients [78]. Taken together, these data suggest the normal vascular response is impaired in the aging kidney, and this contributes to the increased AKI.

### 5.2. Oxidative Stress

The data regarding the role of antioxidants in age-dependent AKI is ambiguous. Although glutathione S-transferase (GST) activity is lower (45% of control) in aged rat kidneys compared to middle-aged and young rats, renal glutathione (GSH) levels are not decreased in the aging rat kidney [40]. We have confirmed that basal GSH levels are not lower in the aging kidney, but levels are depleted more rapidly when challenged with acute stress [44]. Several studies suggest that deficiencies within the antioxidant defense systems may play a role in the aging kidney. A decrease in catalase levels (41% of young) was seen in male Wistar rats at 15 months as compared to 10 and 2.5 month rats; no difference was seen at 10 months as compared to 2.5 [104]. Total plasma antioxidant potential is reduced in aging rats; this may explain the marked increase in oxidative stress (8-hydroxy-2'-deoxyguanosine levels) in the kidney following I/R injury in aged animals [105]. In a study comparing young (3 months), middle-aged (12 months) and aged male (24 months) Wistar rats, I/R injury (as assessed by inulin clearance as a measure of GFR) was more severe in the middle-aged and aged rats [106]. Interestingly, lipid peroxidation was elevated at baseline in middle-aged and aged rats and 30 days supplementation with vitamin E attenuated I/R injury in these rats [106]. The vitamin E supplementation, however, could represent a sub-chronic adaptation, rather than a direct protective effect against acute injury. Furthermore, superoxide dismutase (SOD) attenuated I/R injury in aged rats to a greater extent than the young ones [90]. The induction of hemeoxygenase-1 (HO-1) was blunted in aging mice (12 months old) following I/R injury and the injury was worse in the aging mice [107]. Interestingly, HO-1 was localized to interstitial macrophages, suggesting that they may have a renoprotective role. Overexpression of Sirt1, a NAD-dependent protein deacetylase, in the proximal tubule cell maintains peroxisome number and reduces renal reactive oxygen species levels which rescued cell apoptosis induced by cisplatin [108]. Downregulation of Klotho gene, an anti-aging gene expressed in the distal convoluted tubules, was observed in aging kidney. This was associated with increased susceptibility to oxidative stress via activation of the insulin growth factor-1(IGF-1) pathway [31]. Taken together, these data suggest that decreased antioxidants and increased oxidative stress may play a role in age-dependent AKI; however, significant work remains to elucidate the specific pathways, as well as potential cell-specific effects.

### 5.3. Mitochondria/Apoptosis

Increased cell death due to apoptosis is an intriguing hypothesis for enhanced cell injury with aging, as it represents a convergence between chronic renal dysfunction due to tubular loss and increased AKI in the aging kidney. As such, an increase in salicylate-induced nephrotoxicity was seen in 12 month old rats, as compared to young controls (3 months) [109]. Interestingly, there was evidence of an increased sensitivity of the mitochondria in the proximal tubular epithelial cells to injury. Further evidence for a role of the mitochondria comes from a study of I/R injury in aged (27 months) Wistar rats [110]. Tubular cell apoptosis was increased in aged rats as compared to young controls; basal levels of cytosolic cytochrome C, active caspase-3/9 were elevated in the aging kidney and the up-regulation following I/R injury of caspase-3/9 was increased in aged rats [111]. Increased expression of Bax, a pro-apoptotic protein, caspase-3/9 and cytochrome C in the aging kidney have been observed in other studies [112,113]. The expression of Bcl-2, which is an apoptosis inhibitor, however, is decreased in aged rat kidney [112]. Moreover, the expression of p21, a cyclin-independent kinase (CDK) inhibitor, which induces apoptosis is increased in aged rats [114]. Higher levels of p53 and p21 were expressed in the aging male C57Bl/6 mice after bilateral renal I/R-induced AKI [46]. Therefore, increased apoptotic cell death could account, in part, for the increased AKI in the aging kidney.

### 5.4. Autophagy

Autophagy is an evolutional conserved process to recycle damaged organelles [115,116]. Recent literature suggests a protective role for autophagy in both I/R-induced and toxicant-mediated AKI [117]. The autophagic removal of damaged mitochondria in the mitochondria-rich proximal tubule cells plays a critical role in protecting against AKI [117]. In the aging male Fischer 344 rat, Atg7 was downregulated, as was LC3, indicating that autophagic function is decreased [118]. In the aging male C57Bl/6 mice, autophagic activity was also diminished [116]. Cui *et al.* demonstrated that autophagy is not induced during ischemic stress by renal proximal tubule cells in the aging kidney, and argued that this contributes to the development of AKI [118]. Pharmacologic induction of autophagy by administrating the mTOR inhibitor, rapamycin or temsirolimus, facilitated renal recovery from AKI during endotoxemia in aged mice [116,119] and pharmacologic inhibition of autophagy with either bafilomycin or 3-methyladenine enhanced cisplatin-induced renal tubular cell death [120]. More severe morphologic derangements and greater elevation in serum creatinine levels were observed in proximal tubule-specific autophagy-deficient mice in response to I/R or cisplatin injury [117,119,121]. These data suggest that autophagy, and its role in AKI, represent a novel and fruitful area to be explored in the aging kidney.

### 5.5. Inflammation

Aging is associated with chronic inflammation [122,123] which is characterized by progressive accumulation of lymphocytes and macrophages in renal interstitium [121]. Since inflammation is a well-established mediator of AKI [124,125], a role for inflammation in age-related AKI is highly plausible. A higher influx of lymphocytes and macrophages was detected, accompanied by an increase in tumor necrosis factor-α (TNF-α) and monocyte chemoattractant protein-1 (MCP-1) mRNA expression in aged kidney following transient ischemic injury [46]. In microarray-based analyses of the aging kidney, both human [126], and rat [44], a large percentage of the up-regulated genes were linked to inflammation. In another microarray-based study, the aging kidney was shown to be marked by inflammatory cell infiltration, as demonstrated by the dramatic increase in expression of B- and T-cell specific genes [127]. In both the human and rat microarrays, MMP-7 was shown to be significantly up-regulated; in addition to its proteolytic function, MMP-7 has a pro-inflammatory role [128]. The role of specific pro-inflammatory mediators in the aging kidney, and their relationship to AKI, is an area that will demand future attention. Importantly, AKI-induced inflammation can also promote renal senescence. AKI induces inflammatory cytokines that enhance extracellular matrix deposition, fibrosis and cell apoptosis [65,129]. Recently, a transcriptomics study unveiled AKI induces TWEAK engagement of Fn14 which promotes inflammation via secretion of CXCL16 in renal tubular cells and suppression of anti-aging hormone Klotho through an NF-κB dependent manner, thereby mechanistically linking AKI with aging [130].

### 5.6. Repair

Given that the clinical evidence suggests that AKI is associated with delayed, or decreased, repair in the elderly, there has been a recent surge of work examining aging and kidney repair [31,112]. Baraldi *et al.* suggested that complete recovery was reduced in the elderly patients [28]. Arora *et al.* demonstrated that recovery from AKI, as determined by time to normalization of serum creatinine, was three-times as long in elderly (mean 67.1) compared to young (32.3) patients (32 *vs.* 11.4 days, respectively) [37]. Schmitt *et al.* examined data from 17 studies of AKI and found that a higher percentage of surviving elderly (>65 years) patients did not recover renal function as compared to younger patients; the RR was 1.28 (95% confidence interval of 1.06–1.55) [112]. Fortunately, data from animal studies are in agreement with the clinical findings. In a seminal study, Cantley and coworkers demonstrated that zinc-α(2)-glycoprotein (Zag), an inhibitor of epithelial cell proliferation, is elevated (6.4-fold) in proximal tubular epithelial cells from aged mice (19–24 months) [131]. Overexpression of Zag decreased proliferation of proximal tubular epithelial cells *in vitro* and, importantly, knockdown of Zag in the kidneys of aged mice using siRNA increased proliferation following I/R injury *in vivo*. Additionally, Zag knockdown increased peritubular deposition of collagen IV which was hypothesized to attenuate recovery.

Several mechanisms may underlie the decreased repair potential of the aging kidney. Miya *et al.* have shown that decrease in DNA synthesis was seen following I/R injury in the aging kidney [87]. The expression of P16^INK4A^, a CDK4/6 inhibitor that can block cell-cycle, is increased in epithelial and interstitial cells of aging human and mouse kidneys [132,133]. Ablation of P16^INK4A^ resulted in proliferation and improved regeneration following ischemic injury [134]. Moreover, the expression of p21, another CDK inhibitor which inhibits cell proliferation, is increased in aged rats [114]. The increased expression of p21 and P16^INK4A^ can be accompanied by the accumulation of Cyclin D1 which might reflect an abnormal G1-S transition [135,136]. Furthermore, both p21 and P16^INK4A^ enhance telomere shortening, promoting the senescence of renal tubules [137]. Telomere shortening was also observed in the aging human kidney and is more pronounced in cortex [138].

Growth factors are critical mediators in kidney repair [139]. The expression of factors promoting angiogenesis, cell proliferation and cell recruitment such as vascular endothelial growth factor (VEGF), epidermal growth factor (EGF) and insulin-like growth factor (IGF)-1, decline in the aging kidney [140,141,142]. By contrast, the expression of pro-fibrotic growth factors, including TGF-β1, connective tissue growth factor (CTGF) and integrin-linked kinase (ILK) are increased in the aging kidney [114,143]. These data demonstrate an imbalance of growth factor expression which favors the development of tubulointerstitial fibrosis and an anti-angiogenic environment which could result in a deficient repair process in the aging kidney [144].

Another factor in the decreased ability of the aging kidney to repair following injury may be the decreased expression of components of the cadherin/catenin complex that mediates cell-cell adhesion in the proximal tubule. Our laboratory has demonstrated that the expression of α(E)-catenin is decreased in the aging kidney [145,146]. Given the importance of this complex in establishing cell polarity and regulating the actin cytoskeleton, this deficiency may inhibit the complete recovery of the tubular epithelium in the aging kidney. This was supported by our recent study which demonstrated that loss of α(E)-catenin expression leads to down-regulation of BMP-7 and *N*-cadherin, decreasing repair in renal tubule epithelial cells due to alterations in cell migration [145,146].

Finally, Bonventre and coworkers have shown that a G2/M cell cycle arrest shifts the outcome from repair to fibrosis following AKI [147]. Mechanistically, the G2/M arrest is associated with activation of JNK, which then upregulates profibrotic cytokines, including TGF-β1 and CTGF; interestingly these are overxpressed in the aging kidney [114,143]. Rescue from this arrest, using pharmacological approaches, attenuates the fibrotic response. These results demonstrate a definitive link between deficient repair and progression to CKD in the kidney.

## 6. Conclusions

Renal aging is a complex multifactorial process which predisposes to AKI in the elderly population. Unfortunately, there is no effective therapy currently available for AKI. It is clear, however, that the increased susceptibility of the aging kidney to injury is complex and, most likely, cannot be accounted for by a single mechanism. This is highlighted by the findings that injury is increased, while repair is decreased in the aging kidney, and within each of these pathways there are many converging mechanisms at play. Hopefully, the deeper understanding of all the mechanisms underlying AKI in elderly patients will lead to progression in the development of preventive and protective interventions that decrease the dialysis-requiring AKI and potentiate the resolution of AKI.
